# Deficient activation of CD95 (APO-1/ *Fas*)-mediated apoptosis: a potential factor of multidrug resistance in human renal cell carcinoma

**DOI:** 10.1054/bjoc.2000.1155

**Published:** 2000-06

**Authors:** U Ramp, M Dejosez, C Mahotka, B Czarnotta, T Kalinski, M Wenzel, I Lorenz, M Müller, P Krammer, H E Gabbert, C D Gerharz

**Affiliations:** Institute of Pathology, Heinrich Heine University, Moorenstrasse 5, Duesseldorf, D-40225, Germany; Institute of Transfusion Medicine, Heinrich Heine University, Duesseldorf, Germany; Department of Internal Medicine IV, University Hospital, Heidelberg, Germany; German Cancer Research Center (DKFZ), Heidelberg, Germany

**Keywords:** CD95 (APO-1/Fas), apoptosis, anticancer drugs, multidrug resistance, renal cell carcinoma

## Abstract

The pronounced resistance of human renal cell carcinoma (RCC) to anticancer-induced apoptosis has primarily been related to the expression of P-glycoprotein and effective drug detoxification mechanisms. Because the CD95 system has recently been identified as a key mediator of anticancer drug-induced apoptosis, we analysed the contribution of the CD95 system to chemotherapy-induced apoptosis in four newly established RCC cell lines. Here, we demonstrate that all RCC cell lines expressed CD95-receptor and -ligand. Exposure to *agonistic* anti-CD95 antibodies resulted in induction of apoptosis and significant (*P*< 0.05) reduction of cell number in three out of four cell lines, indicating that the essential components for CD95-mediated apoptosis were present and functionally intact in the majority of these RCC cell lines. Moreover, treatment of cultures with bleomycin or topotecan, a novel topoisomerase I inhibitor with little substrate affinity for P-glycoprotein, led to induction of apoptosis and significant (*P*< 0.05) dose-dependent reduction of cell number in all RCC cell lines. Both anticancer drugs also induced upregulation of CD95 ligand expression in all cell lines. Additionally, augmentation of CD95 receptor expression was found in three RCC cell lines, including one p53-mutated cell line, whereas another p53-mutated cell line showed no or only a weak CD95 receptor upregulation after exposure to topotecan or bleomycin, respectively. Despite this upregulation of CD95 receptor and ligand, *antagonistic* antibodies directed against CD95 receptors or ligands could not inhibit induction of apoptosis by topotecan and bleomycin in any cell line. Thus, although a functionally intact CD95 signalling cascade is present in most RCC cell lines, the anticancer drugs topotecan and bleomycin that induce upregulation of CD95 receptor and ligand fail to effectively activate CD95-mediated apoptosis. This deficient activation of CD95-mediated apoptosis might be an important additional factor for the multidrug resistance phenotype of human RCCs. © 2000 Cancer Research Campaign


British Journal of Cancer (2000) 82(11), 1851–1859
© 2000 Cancer Research Campaign
DOI: 10.1054/ bjoc.2000.1155, available online at http://www.idealibrary.com on IDEAL®

### Deficient activation of CD95 (APO-1/*Fas*)-mediated apoptosis: a potential factor of multidrug resistance in human renal cell carcinoma

**U Ramp[1], M Dejosez[1], C Mahotka[1], B Czarnotta[1], T Kalinski[1], M Wenzel[1], I Lorenz[2], M Müller[3], P Krammer[4], HE Gabbert[1] and CD Gerharz[1]**

[1]Institute of Pathology, Heinrich Heine University, Moorenstrasse 5, D-40225 Duesseldorf, Germany; [2]Institute of Transfusion Medicine, Heinrich Heine University, Duesseldorf, Germany; [3]Department of Internal Medicine IV, University Hospital, Heidelberg, Germany; [4]German Cancer Research Center (DKFZ), Heidelberg, Germany

**Summary** The pronounced resistance of human renal cell carcinoma (RCC) to anticancer-induced apoptosis has primarily been related to the expression of P-glycoprotein and effective drug detoxification mechanisms. Because the CD95 system has recently been identified as a key mediator of anticancer drug-induced apoptosis, we analysed the contribution of the CD95 system to chemotherapy-induced apoptosis in four newly established RCC cell lines. Here, we demonstrate that all RCC cell lines expressed CD95-receptor and -ligand. Exposure to *agonistic* anti-CD95 antibodies resulted in induction of apoptosis and significant ($P$ < 0.05) reduction of cell number in three out of four cell lines, indicating that the essential components for CD95-mediated apoptosis were present and functionally intact in the majority of these RCC cell lines. Moreover, treatment of cultures with bleomycin or topotecan, a novel topoisomerase I inhibitor with little substrate affinity for P-glycoprotein, led to induction of apoptosis and significant ($P$ < 0.05) dose-dependent reduction of cell number in all RCC cell lines. Both anticancer drugs also induced upregulation of CD95 ligand expression in all cell lines. Additionally, augmentation of CD95 receptor expression was found in three RCC cell lines, including one p53-mutated cell line, whereas another p53-mutated cell line showed no or only a weak CD95 receptor upregulation after exposure to topotecan or bleomycin, respectively. Despite this upregulation of CD95 receptor and ligand, *antagonistic* antibodies directed against CD95 receptors or ligands could not inhibit induction of apoptosis by topotecan and bleomycin in any cell line. Thus, although a functionally intact CD95 signalling cascade is present in most RCC cell lines, the anticancer drugs topotecan and bleomycin that induce upregulation of CD95 receptor and ligand fail to effectively activate CD95-mediated apoptosis. This deficient activation of CD95-mediated apoptosis might be an important additional factor for the multidrug resistance phenotype of human RCCs. © 2000 Cancer Research Campaign

**Keywords:** CD95 (APO-1/Fas); apoptosis; anticancer drugs; multidrug resistance; renal cell carcinoma

Human renal cell carcinoma (RCC) is known to be largely resistant to conventional chemotherapy, resulting in an extremely poor prognosis once the tumour has metastasized and is beyond the reach of curative surgery (Mulders et al, 1997). Resistance to chemotherapy has been explained by multiple mechanisms, including the multidrug resistance phenotype (Baldini, 1997; Mulders et al, 1997) or defects in apoptotic pathways (Hetts, 1998). Recent evidence indicates that CD95-mediated apoptosis might also effectively determine the response of cancer cells to cytotoxic drugs (Friesen et al, 1996; 1997; Strand et al, 1996; Fulda et al, 1997; Krammer, 1997; Müller et al, 1997).

CD95 is a 45 kDa type I transmembrane receptor that confers an apoptotic signal to sensitive cells upon trimerization by its ligand CD95L, a type II membrane protein (Nagata, 1994). Following activation of CD95-receptors, a death signal is generated that leads to the activation of a cascade of cysteine proteases (caspases) and finally to apoptosis (Kischkel et al, 1995). This apoptotic pathway has originally been explored within the immune system only. However, recent evidence indicates that the CD95 pathway is also involved in drug-induced apoptosis of tumour cells (Krammer, 1997). Upregulation of CD95-ligand and/or -receptor has thus been demonstrated in drug-sensitive leukaemias, hepatocellular carcinomas and neuroblastomas upon treatment with various anticancer drugs, and, most importantly, blocking of CD95 receptor activation by antagonistic antibodies effectively inhibited drug-induced apoptosis (Friesen et al, 1996; Fulda et al, 1997; Müller et al, 1997). In contrast, drug-resistant leukaemia cells failed to upregulate CD95-ligand expression and to activate the CD95-pathway (Friesen et al, 1997). Remarkably, upregulation of CD95 receptor expression upon anticancer drug-treatment was observed only in p53 wild-type hepatocellular carcinomas, indicating that p53 might also be involved in the regulation of CD95-mediated apoptosis (Müller et al, 1997; 1998). These observations suggested that chemotherapeutic agents induce apoptosis via CD95-dependent pathways and that resistance of tumours to chemotherapy might also be related to deficient activation of the CD95 system.

Although CD95 receptor expression and CD95-mediated apoptosis have clearly been demonstrated in human RCCs (Caignard et al, 1996; Nonomura et al, 1996; Tomita et al, 1996; Horie et al, 1997; Miyake et al, 1998), the role of CD95-mediated apoptosis for chemoresistance in human RCCs has yet to be established. The aim of the present investigation, therefore, was to study the

*Received 20 August 1999*
*Revised 3 January 2000*
*Accepted 9 January 2000*

*Correspondence to:* CD Gerharz functional contribution of the CD95 system to chemotherapy-induced apoptosis in human RCCs. To this end, we exposed newly established human RCC cell lines of different histological types to topotecan, a novel topoisomerase I inhibitor with little substrate affinity for P-glycoprotein (Hendricks et al, 1992; Sorensen et al, 1995), or to the DNA-damaging antibiotic bleomycin, which has been shown to act via the CD95 system (Müller et al, 1997).

### MATERIALS AND METHODS

### Cells and culture

All four cell lines used in this study were derived from typical representatives of the clear cell (clearCa-3, -6, -17) and chromophilic/papillary (chromphi-3) types of RCC (Gerharz et al, 1993; 1994; 1995; 1996). The cell lines were maintained with Dulbeccos's modified Eagle's medium (DMEM, Gibco, FRG), supplemented with 10% heat-inactivated fetal calf serum (FCS), penicillin and streptomycin (= standard growth medium) and cultivated at 37°C in an atmosphere with 5% $CO_2$.

### Analysis of p53 mutations

Extraction of genomic DNA was performed using the QIAmp Tissue Kit (Qiagen, FRG) according to the manufacturer's protocol. For amplification of p53 exons 5 to 8, the following oligonucleotide primers were used: Exons 5 and 6: (forward) 5′-TTC CTC TTC CTG CAG TAC TC-3′; (reverse) 5′-ATG TGC AAA CCA GAC CTC AG-3′. Exons 7 and 8: (forward) 5′-GTG TTG TCT CCT AGG TTG GC-3′; (reverse) 5′-AAG TGA ATC TGA GGC ATA AC-3′. Each amplification reaction was carried out in a total volume of 50 µl containing 200 ng of genomic DNA, 10 pmol of each primer, 10 nM of each dNTP, 2 U Taq polymerase and PCR-reaction-buffer (Sigma, FRG). After an initial denaturation step at 94°C for 2 min, 35 cycles of denaturation at 94°C for 30 s, annealing at 50°C for 40 s, and extension at 72°C for 1 min, as well as a last delay at 72°C for 10 min, were performed. The PCR-products were purified from surplus oligonucleotides using Microspin S-300 columns (Pharmacia, FRG). The purified PCR-products were prepared for automatic sequencing using the ABI-Prism BigDye Terminator Cycle Sequencing Kit (Perkin Elmer, FRG) according to the manufacturer's protocol. Sequence analysis was carried out with the sense (5′) and the antisense (3′) primer using an ABI-Prism 310 sequencer (Perkin Elmer). p53 mutations were verified by an independent PCR-amplification of genomic DNA followed by a repeated sequencing.

### RNA extraction

Total cellular RNA was isolated from RCC cell lines using either the RNeasy kit (Qiagen) for the detection of CD95 receptor mRNA, or the caesium chloride ultracentrifugation method for the detection of CD95 ligand and p21 mRNA. RNA concentration was measured by photometry at 260 nm.

### RT-PCR analysis

For monitoring CD95 receptor and CD95 ligand expression, reverse transcription was performed using an RT-kit (Stratagen, FRG), 2 µg (CD95 receptor) or 5 µg (CD95 ligand) of total cellular RNA and oligo(dT) (CD95 receptor) or random primer (CD95 ligand) as RT-primer. The amplification mixture with a final volume of 50 µl was composed as follows: 5 µl of the cDNA template, 10 pM $\mu l^{-1}$ (CD95 receptor) or 50 pM $\mu l^{-1}$ (CD95 ligand) of the gene-specific primers, 200 µM (CD95 receptor) or 100 µM (CD95 ligand) of each dNTP and 1.5 U Taq-polymerase (Sigma).

For the amplification of CD95 receptor, the following primers were used (Cascino et al, 1995): (forward) 5′ CAG AAC TTG GAA GGC CTG CAT C 3′; (reverse) 5′ GGA CTT TGT CAC CGT TAT TTA 3′. These oligonucleotides amplified the transmembranous CD95 receptor (510 bp) as well as a splice variant with a 63 bp deletion of the transmembranous region encoding the soluble CD95 receptor (447 bp). The initial denaturation step at 94°C for 4 min was followed by 25 cycles of denaturation for 30 s, annealing at 57°C for 30 s, extension at 72°C for 40 s, and a final extension step at 72°C for 10 min. The PCR products were separated on a 1.5% agarose gel and the ratio between both splice variants was quantified by densitometry (Biometra, FRG) in three independent experiments.

For the amplification of CD95 ligand, the following primers were used (Peter et al. 1995): (forward) 5′ ATA GGA TCC ATG TTT CTG CTC TTC CAC CTA CAG AAG GA 3′; (reverse) 5′ ATA GAA TTC TGA CCA AGA GAG AGC TCA GAT ACG TTG AC 5′. The initial denaturation step at 94°C for 4 min was followed by 35 cycles of denaturation for 1 min, annealing at 54°C for 1 min, extension at 72°C for 1 min, and a final extension step at 72°C for 10 min. Identity of the amplification products was confirmed by direct sequencing (data not shown).

### Northern blot analysis

Northern blot analysis was carried out for 20 µg RNA of each sample under denaturating conditions with a 1% formaldehyde gel. After transfer of the RNA to the Nylon membrane (Amersham, FRG), the RNA was hybridized with a specific p21 cDNA probe generated by RT-PCR (primers: forward: 5′ CAG TGG ACA GCG AGC AGC TG 3′; reverse: 5′ ATC TGT CAT GCT GGT CTG CC 3′; 35 cycles of denaturation for 30 s, annealing at 58°C for 30 s, extension at 72°C for 40 s). The DNA was labelled by incorporation of $^{32}$P-dCTP using the oligo-labelling kit (Pharmacia). Hybridization, stringent washing procedures and radiography were carried out as previously described (Ramp et al, 1997). All experiments were done twice with different Nylon membranes and the results obtained were reproducible.

### Flow cytometric analysis

The expression of CD95 receptor and CD95 ligand was assessed by fluorescence-activated cytometry carried out in a FACScan® flow cytometer (Becton Dickinson, FRG). After 24 h in standard growth medium, RCC cells were incubated for another 24, 48, and 72 h in growth medium supplemented with topotecan (1 µg $ml^{-1}$) or bleomycin (300 µg $ml^{-1}$). As control, cells were maintained in standard growth medium. 1 µg of the FITC-labelled monoclonal antibodies (CD95 receptor-FITC: monoclonal $IgG_1$ (mouse) antibody UB2, Immunotech, FRG; CD95 ligand-FITC: monoclonal $IgG_{2a}$ (rat) antibody H11, Alexis, FRG) was added to each tube with $1 \times 10^5$ cells in a final reaction volume of 200 µl and incubated for 20 min at 4°C. Two washing steps were performed

 *© 2000 Cancer Research Campaign*

before measuring the cells. As controls, the FITC-labelled isotype antibodies mouse-$IgG_1$ (Immunotech) for CD95 receptor and rat-$IgG_{2a}$ for CD95 ligand (Pharmingen, FRG) were used. All experiments were done twice and the results obtained were reproducible.

### Induction and quantification of apoptosis

$1 \times 10^4$ cells were seeded in each chamber of 8-chamber slides (Nunc, FRG). After 24 h, the cells were treated with topotecan (0.01, 0.1, 1, or 10 μg $ml^{-1}$) *or* bleomycin (3, 30 or 300 μg $ml^{-1}$) *or* CD95 receptor-activating CH11 antibody (0.5 μg $ml^{-1}$, Immunotech) (Natoli et al, 1995; Keane et al, 1996; O'Connell et al, 1996; Strand et al, 1996) for another 48 h. For IFN-γ pretreatment, tumour cells were exposed to IFN-γ (100 U $ml^{-1}$; Biosource, FRG) 24 h after seeding on 8-chamber slides. After another 48 h, the growth medium was substituted by medium supplemented with the CH11 antibody and cells were cultivated for another 48 h. As a control, tumour cells were exposed to either standard growth medium or growth medium supplemented with IFN-γ (100 U $ml^{-1}$). The number of apoptotic cells per $1 \times 10^3$ cells was determined by light microscopical counting of haematoxylin-eosin (HE)-stained cells showing the typical morphological signs of apoptosis, i.e. chromatin condensation and/or fragmentation into apoptotic bodies. Counting of apoptotic cells was performed in two independent experiments and the data presented are the mean out of these experiments. Specific apoptotic death (SAD) was calculated as the frequency of apoptotic cells after exposure to topotecan *or* bleomycin *or* CH11 *or* CH11 + IFN-γ minus the frequency of apoptotic cells in standard growth medium.

### Assessment of cell number

Tumour cells in the exponential growth phase were transferred to microwell plates (Gibco) at $1 \times 10^4$ cells per well in 0.1 ml standard growth medium. After 24 h cells were exposed to topotecan (0.01, 0.1, 1, or 10 μg $ml^{-1}$) *or* bleomycin (3, 30, or 300 μg $ml^{-1}$) *or* CD95 receptor-activating CH11 antibody (0.5 μg $ml^{-1}$, Immunotech) in growth medium. As a control, tumour cells were cultivated in standard growth medium. The plates were incubated for another 48 h (topotecan or bleomycin) or 72 h (CH11) at 37°C and 5% $CO_2$. For IFN-γ pretreatment, tumour cells were exposed to IFN-γ (100 U $ml^{-1}$) for 48 h, and then incubated for another 72 h in growth medium supplemented with CH11 (0.5 μg $ml^{-1}$). As a control for the effects of IFN-γ on cell number, tumour cells were exposed to IFN-γ for 48 h and then incubated for another 72 h in standard growth medium. The number of viable tumour cells was then analysed using the colorimetric MTT assay (Mosman, 1983) and measured on a spectrophotometric plate reader (Titertek Multiscan, FRG) at 570 nm.

The data presented are the mean ± standard deviation from 24 replicate wells per microtitre plate and three independent experiments per cell line. The data were statistically evaluated by an analysis of variance with two independent factors. The 50% inhibitory drug concentration ($IC_{50}$ value) was statistically determined by SSPS (probit analysis).

Interactions between CH11 and topotecan were analysed by the fractional inhibition method as follows: when expressed as the fractional inhibition cell viability, additive inhibition produced by both inhibitors (i) occurs when $i_{1,2} = i_1 + i_2$; synergism when $i_{1,2} > i_1 + i_2$; and antagonism when $i_{1,2} < i_1 + i_2$ (Webb, 1963).

### Blockade of CD95 signalling by antibodies

To analyse the functional contribution of the CD95 system to anticancer drug-induced apoptosis in RCC, we used antagonistic antibodies directed against CD95 receptors (i.e. $F(ab)'_2$-anti-APO-1 antibody fragments made by PK's laboratory) (Friesen et al, 1996; Fulda et al, 1997; 1998; Müller et al, 1997), or antibodies binding CD95 ligands (i.e. monoclonal 4H9 antibodies, Immunotech) (Nakajima et al, 1998). Both antibodies competitively inhibit binding of CD95 ligand to the CD95 receptor, resulting in a blockade of the CD95 signalling pathway.

RCC cells were exposed to topotecan (1 μg $ml^{-1}$) *or* bleomycin (300 μg $ml^{-1}$) as single agents *or* in combination with the $F(ab)'_2$-anti-APO-1 antibody fragments (0.1 or 1 μg $ml^{-1}$) *or* 4H9 antibodies (0.1 or 1 μg $ml^{-1}$). RCC tumour cells were then incubated for 48 h and MTT assays were performed as described above.

### RESULTS

### Anticancer drugs induce apoptotic cell death in human RCCs

Exposure to topotecan or bleomycin resulted in induction of apoptosis, as became evident from light microscopic inspection of cell cultures (Figure 1). The determination of specific apoptotic death (SAD) confirmed a dose-dependent increase of apoptotic cell

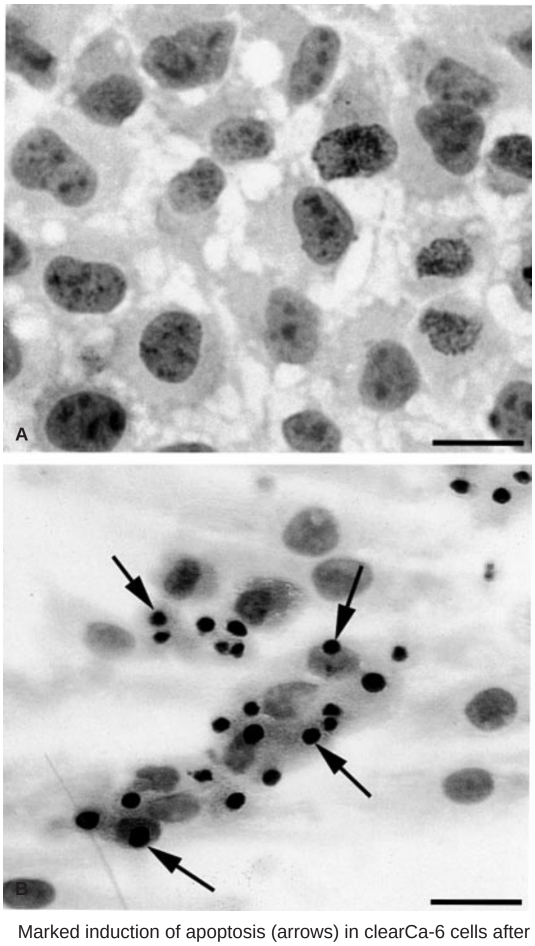

**Figure 1** Marked induction of apoptosis (arrows) in clearCa-6 cells after exposure to topotecan (**B**) when compared to the control (**A**) (bar = 50 μm)

© 2000 Cancer Research Campaign 

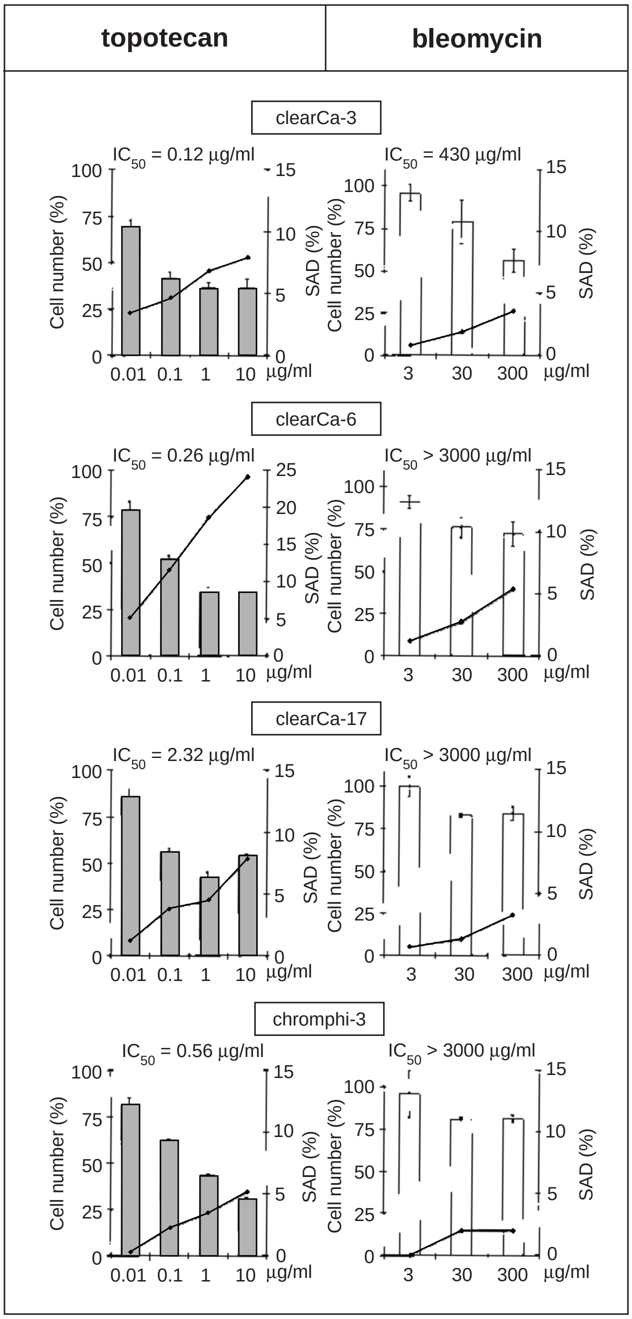

**Figure 2** Effects of topotecan or bleomycin on apoptosis and proliferation. Exposure to topotecan or bleomycin resulted in an increase of specific apoptotic death (SAD) as well as a significant ($P < 0.05$) dose-dependent reduction of cell number in all RCC cell lines (vertical bars indicate standard deviations)

death in all RCC cell lines (Figure 2), the response being most pronounced in clearCa-6.

Induction of apoptosis was paralleled by a significant ($P < 0.05$) dose-dependent reduction of cell number, the $IC_{50}$ values ranging from 0.12 µg $ml^{-1}$ to 2.32 µg $ml^{-1}$ for topotecan and from 430 µg $ml^{-1}$ to more than 3000 µg $ml^{-1}$ for bleomycin (Figure 2).

It is noteworthy that the effects of topotecan became evident already at clinically relevant concentrations (< 1 µg $ml^{-1}$) (Herben et al, 1996), whereas the $IC_{50}$ values of bleomycin were at least 100 times higher than the peak plasma concentration achievable during chemotherapy in vivo (Scheithauer et al, 1986).

### Anticancer drugs augment expression of CD95 receptors and ligands in human RCCs

As shown by RT-PCR (Figure 3) and flow cytometry (Figures 4 and 5), all RCC cell lines showed constitutive expression of CD95 receptor and ligand in standard culture medium.

Exposure to topotecan (1 µg $ml^{-1}$) resulted in a time-dependent increase in the expression of CD95 receptor *and* ligand in three out of four cell lines, as revealed by flow cytometry (Figures 4 and 5). The maximum expression was observed 72 h after exposure to topotecan and only minor differences in the extent of induction became evident between these cell lines. In contrast, clearCa-17 cells showed only a weak increase of CD95 ligand expression and no concomitant upregulation of CD95 receptor expression (Figures 4 and 5), although this cell line had also responded with an increase of apoptotic cell death.

Exposure to bleomycin (300 µg $ml^{-1}$) resulted in an augmentation of CD95-receptor *and* -ligand expression in all cell lines with a maximal induction after 48 h (clearCa-3, -17, and chromphi-3) or 72 h (clearCa-6) (Figures 4 and 5).

### Anticancer drug-induced upregulation of CD95 receptor and ligand occurs in p53-mutated RCCs

Because p53 has been reported to be involved in the augmentation of CD95 receptor expression in hepatocellular carcinomas after exposure to anticancer agents (Müller et al, 1998), we additionally defined the mutational status of p53 in our RCC cell lines.

Sequencing of p53 exons 5–8, which are the most commonly affected hot-spot regions for p53 mutations in human cancer (Harris and Hollstein, 1993; Reiter et al, 1993), revealed no mutations in clearCa-3 and chromphi-3. In contrast, isolated point mutations were found in clearCa-6 and clearCa-17 (Table 1).

Whereas clearCa-17 showed no (topotecan) or a weak (bleomycin) increase of CD95 receptor expression, clearCa-6 responded with marked upregulation of CD95 receptor upon exposure to both anticancer drugs despite p53 mutation. Moreover, upon exposure to topotecan, clearCa-6 was still able to transcriptionally activate the expression of p21 (Figure 6), known to be directly regulated by p53 (El-Deiry et al, 1993; Waldman et al, 1995).

### Anticancer drug-induced apoptosis is not mediated via the CD95 pathway in human RCCs

The augmentation of CD95 receptor and/or ligand expression observed in our RCC cell lines after treatment with topotecan or bleomycin might suggest involvement of the CD95-system in drug-induced cell death. To test this hypothesis, we competitively inhibited the binding of CD95 ligand and hence CD95 receptor activation either by *antagonistic* $F(ab)'_2$-anti-APO-1 antibody fragments or by 4H9 anti-CD95 ligand antibodies. If the apoptotic effects of anticancer drugs were actually mediated via the CD95-system, simultaneous incubation of RCC cells with these antibodies should block the effects of anticancer drugs.

The data obtained in these experiments are summarized in Figure 7, showing that the reduction of cell number observed after exposure to topotecan or bleomycin could *not* be blocked by antibodies directed against CD95 receptors or ligands in any cell line.

These observations clearly show the existence of a CD95-independent pathway for the action of topotecan and bleomycin in all our RCC cell lines.

*© 2000 Cancer Research Campaign*

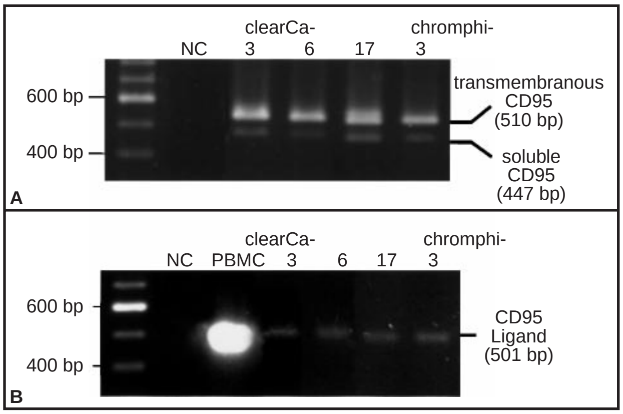

**Figure 3** Constitutive expression of CD95 receptor and ligand. (**A**) Using primers simultaneously amplifying the transcripts of transmembranous and soluble CD95 receptor, RT-PCR demonstrated coexpression of both CD95 receptor forms in all RCC cell lines with a predominance of the trans membranous variant. (**B**) RT-PCR revealed CD95 ligand expression in all cell lines (Concanavalin A-activated peripheral blood mononuclear cells (PBMC) were used as positive control, NC = negative control)

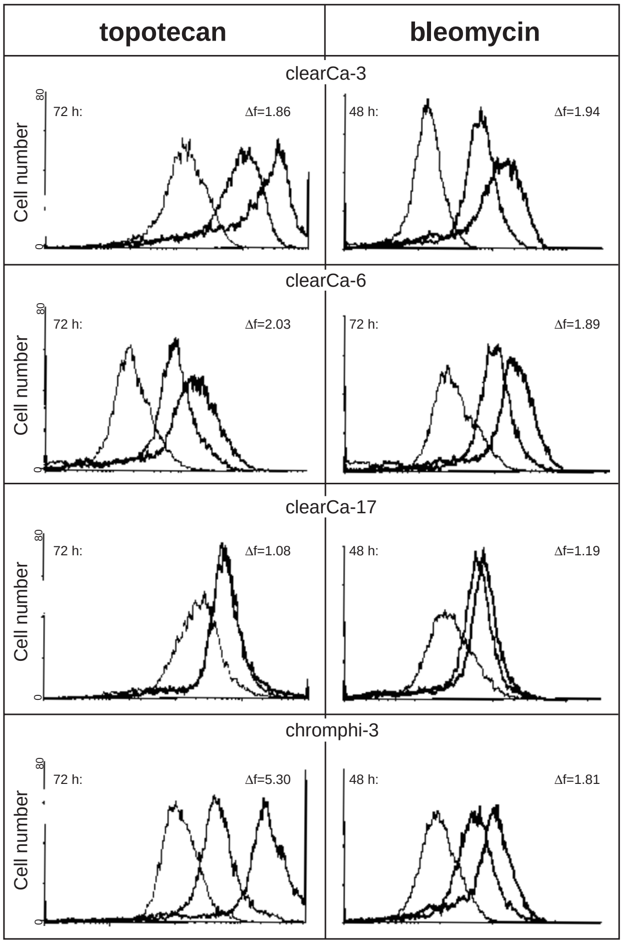

**Figure 4** CD95 receptor expression after exposure to topotecan or bleomycin. Exposure to topotecan (1 μg ml$^{-1}$; thick line) or bleomycin (300 μg ml$^{-1}$; thick line) resulted in increase of CD95 receptor expression in clearCa-3, -6, and chromphi-3 when compared with the control (medium line). No increase of CD95 receptor expression was found in clearCa-17 after exposure to topotecan (thin line = isotope control. Presented is the maximum of CD95 receptor induction. Δf describes the difference of mean fluorescence intensities between drug-exposed tumour cells and the untreated control)

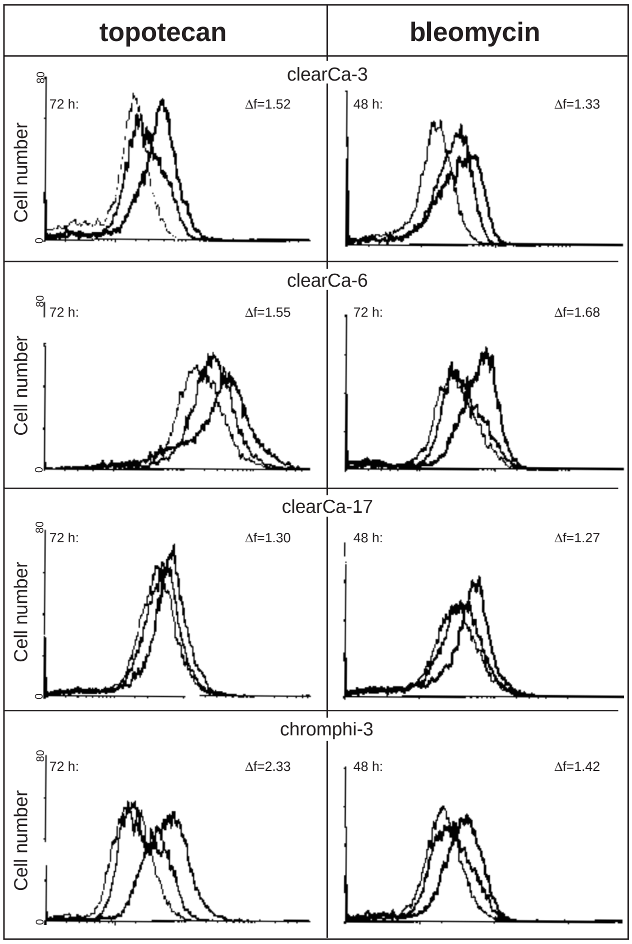

**Figure 5** CD95 ligand expression after exposure to topotecan or bleomycin. Exposure to topotecan (1 μg ml$^{-1}$; thick line) or bleomycin (300 μg ml$^{-1}$; thick line) resulted in increase of CD95 ligand expression in all RCC cell lines when compared with the control (medium line) (thin line = isotype control. Presented is the maximum of CD95 ligand induction. Δf describes the difference of mean fluorescence intensities between drug-exposed tumor cells and the untreated control)

### Essential components for CD95-mediated apoptosis are present in human RCCs

Because the upregulation of CD95 receptor and ligand after exposure to anticancer drugs was not followed by CD95-mediated cell death, we hypothesized that the response to CD95 receptor activation might seriously be disturbed in our RCC cell lines. To test this hypothesis, we used the *agonistic* anti-CD95 antibody CH11 to confront RCC cell lines with a uniform pro-apoptotic signal.

As shown in Table 2, CD95 receptor activation by *agonistic* CH11 antibodies resulted in an increase of specific apoptotic death (SAD) in all RCC cell lines of the clear cell type, which was followed by a significant ($P$ < 0.05) reduction of cell number. Differences between the extent of apoptotic response and reduction of cell number probably reflect differences in the onset and kinetics of CD95-mediated apoptosis in our cell lines. After pretreatment with IFN-γ, which is known to increase CD95 receptor expression (Nonomura et al, 1996), CH11-triggered apoptotic cell death was further augmented, as indicated by the increase of SAD and the reduction of cell number (Table 2). In contrast, chromphi-3 cells proved to be largely resistant against CD95

© 2000 Cancer Research Campaign

**Table 1** p53 status of exons 5 to 8 determined by DNA sequencing

| Cell lines | Wild-type (wt) and mutations (mt) of p53 |
|---|---|
| clearCa | |
| 3 | wt |
| 6 | mt: exon 8, codon 290: CGC (Arg) to CAC (His) |
| 17 | mt: exon 6, codon 213: CGA (Arg) to CTA (Leu) |
| chromphi | |
| 3 | wt |

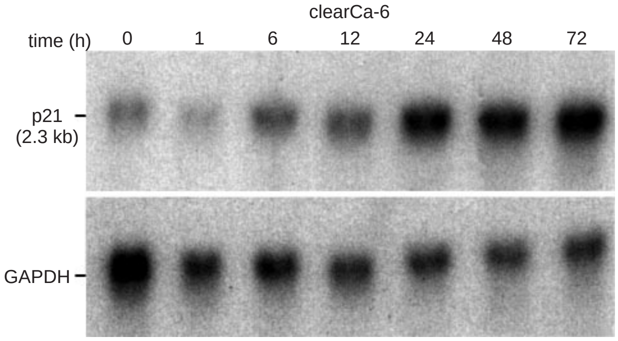

**Figure 6** Upregulation of p21 expression after exposure to topotecan. After exposure to topotecan (1 μg ml$^{-1}$), Northern blot revealed increase of p21 expression in p53-mutated clearCa-6 cells, thereby suggesting functionally intact p53 despite mutation. Hybridization with GAPDH demonstrated the equality of RNA loaded in each lane receptor activation by CH11 antibodies, even after IFN-γ pretreatment. In conclusion, the essential components for CD95-mediated cell death were present and functionally intact in all RCC cell lines of the clear cell type, but not in the chromophilic RCC cell line. Nevertheless, the upregulation of CD95 receptor and ligand induced by topotecan or bleomycin was not sufficient for an effective activation of CD95-mediated apoptosis.

Moreover, we asked whether anticancer drug-induced upregulation of CD95 receptor expression might be exploited for CD95-mediated apoptosis applying CH11 antibodies. To answer this question, clearCa-3 and chromphi-3 cells (which had been least responsive to CH11 antibodies as a single agent) were simultaneously exposed to both topotecan (1 μg ml$^{-1}$) and CH11 (0.5 μg ml$^{-1}$) for 72 h. As shown in Figure 8, both cell lines responded with a marked reduction of cell number. By fractional inhibition analysis, it could be demonstrated that the effects of CH11 and topotecan in combination were synergistic compared with either agent alone.

### DISCUSSION

Ample evidence exists that anticancer drugs exert their effects through induction of apoptosis, irrespective of their primary intracellular targets (Hickman, 1992). Only recently, CD95-triggered apoptosis has been identified as a key mediator of chemotherapy in leukaemias and some solid tumour types (Friesen et al, 1996; Fulda et al, 1997; 1998; Müller et al, 1997). Vice versa, resistance to anticancer drugs in leukaemias was shown to be associated with defects of the CD95-system (Friesen et al, 1997). Up to now, the pronounced resistance of human RCCs to chemotherapy had primarily been related to effective drug elimination by transport proteins such as P-glycoprotein (Baldini, 1997; Mulders et al, 1997) or to drug detoxification by the glutathione/glutathione-S-transferase pathway (Mickisch et al, 1990). Investigations into the function of the CD95 system in RCCs upon treatment with anticancer drugs, therefore, could provide new insights into alternative mechanisms of multidrug resistance in renal cancer and eventually facilitate the design of novel therapeutic concepts.

The data presented here demonstrate that therapeutically relevant concentrations of topotecan induced a marked, dose-dependent increase of apoptosis and a significant ($P < 0.05$) reduction of cell number in all our RCC cell lines. In accordance with previous preclinical and clinical observations (Homma and Aso, 1994), RCC cell lines, however, proved to be largely resistant to bleomycin, showing only a minor reduction of cell number at clinically relevant dose levels (≤ 3 μg ml$^{-1}$).

Response to topotecan was paralleled by a marked increase in the expression of CD95 receptor *and* ligand in three out of four RCC cell lines. It was interesting to note, however, that an almost corresponding upregulation of CD95 receptor and ligand could be induced by bleomycin in all cell lines, although bleomycin had shown far less pronounced effects on cell number when compared to topotecan. These findings are in line with previous reports on anticancer drug-induced upregulation of CD95 receptor and ligand in other tumour models (Friesen et al, 1996; Müller et al, 1997; Fulda et al, 1998). The upregulation of CD95 receptor and ligand upon treatment with anticancer drugs, therefore, is a common reaction pattern in a great variety of different human tumour types, including RCCs.

However, the upregulation of CD95 receptor and ligand observed in our RCC cell lines did not yet prove the actual functionality of the CD95 system in chemotherapy-induced apoptosis.

**Table 2** Cell number and apoptotic response in RCC cell lines after exposure to CH11 (500 ng ml$^{-1}$) *or* IFN-γ (100 U ml$^{-1}$) *or* IFN-γ (100 U ml$^{-1}$) + CH11 (500 ng ml$^{-1}$)

| | | CH11 | | IFN-γ | | IFN-γ + CH11 | |
|---|---|---|---|---|---|---|---|
| Cell lines | BAD (%) | cell number (% of the control) | SAD (%) | cell number (% of the control) | SAD (%) | cell number (% of the control) | SAD (%) |
| clearCa | | | | | | | |
| 3 | 2.6 | 88 ± 5 | 8.3 | 81 ± 4 | 3.3 | 42 ± 2 | 10.3 |
| 6 | 2.1 | 40 ± 4 | 32.1 | 79 ± 10 | 3.5 | 18 ± 1 | 48.9 |
| 17 | 1.7 | 62 ± 3 | 8.4 | 88 ± 6 | 1.2 | 11 ± 1 | * |
| chromphi | | | | | | | |
| 3 | 0.2 | 105 ± 2 | 0 | 93 ± 6 | 0.1 | 91 ± 3 | 0.6 |

Abbreviations: BAD = basal apoptotic death; SAD = specific apoptotic death; * marked response with less than $10^3$ cells per chamber left (cf. Materials and methods)

*© 2000 Cancer Research Campaign*

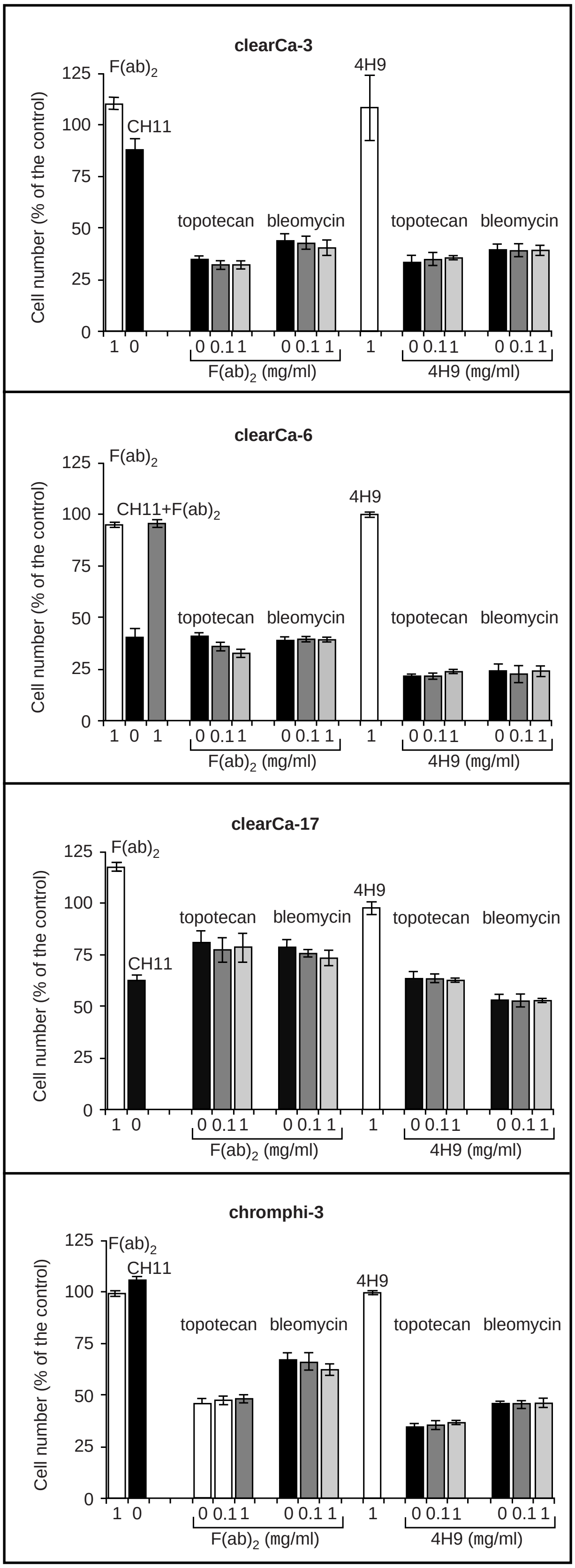

**Figure 7** CD95-antagonistic F(ab)′$_2$-anti-APO-1 antibody fragments or CD95 ligand-binding 4H9 antibodies did not inhibit topotecan- or bleomycin-induced apoptosis. Combined exposure of F(ab)′$_2$-anti-APO-1 antibody fragments (0.1 or 1 μg ml$^{-1}$) *or* 4H9 antibodies (0.1 or 1 μg ml$^{-1}$) with topotecan (1 μg ml$^{-1}$) *or* bleomycin (300 μg ml$^{-1}$) did *not* inhibit reduction of cell number when compared with the effects of topotecan *or* bleomycin as single agents. Exposure to F(ab)′$_2$-anti-APO-1 antibody fragments (1 μg ml$^{-1}$) *or* 4H9 antibodies (1 μg ml$^{-1}$) alone did not affect cell growth in any cell line. CD95 receptor activation by agonistic CH11 antibodies (0.5 μg ml$^{-1}$) resulted in significant ($P < 0.05$) reduction of cell number in all RCC cell lines of the clear cell type. F(ab)′$_2$-anti-APO-1 antibody fragments completely inhibited the reduction of cell number induced by CD95-agonistic CH11 antibodies (0.5 μg ml$^{-1}$) as exemplary shown in clearCa-6.

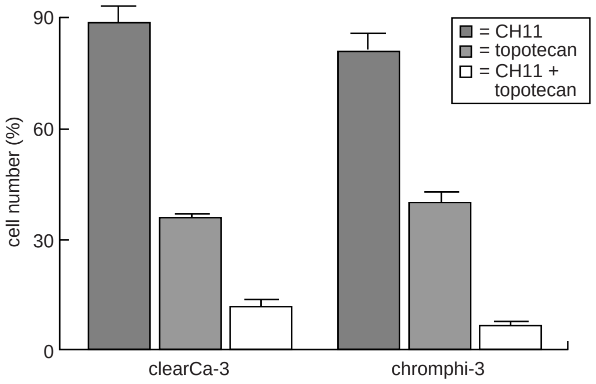

**Figure 8** Synergistic enhancement of CH11-induced apoptosis in clearCa-3 and chromphi-3 by topotecan. Both cell lines were cultured in the presence of CH11 (0.5 μg ml$^{-1}$) or topotecan (1 μg ml$^{-1}$) alone or in combination.

Therefore, we used F(ab)′$_2$-anti-APO-1 antibody fragments or 4H9 anti-CD95 ligand antibodies, which are both known to block the binding of CD95 ligand to its receptor, thereby preventing signal transduction via the CD95 system (Friesen et al, 1996; Fulda et al, 1997; 1998; Müller et al, 1997; Nakajima et al, 1998). In these experiments, the apoptosis induced by topotecan or bleomycin could *not* be inhibited in any RCC cell line. Although not explaining the underlying mechanisms, these results provide convincing evidence that apoptosis induced by these anticancer drugs is mediated via CD95-independent mechanisms in human RCCs. The molecular effector pathways of chemotherapy-induced apoptosis in RCCs, therefore, might differ from those in other solid tumours like carcinomas of liver, colon, and lung, Ewing sarcoma and neuroblastoma. In these tumour types, various anti-cancer drugs including bleomycin have been shown to induce apoptosis – at least in part – via the CD95-system (Fulda et al, 1997; 1998; Müller et al, 1997).

Little is known so far about the underlying mechanisms prohibiting the activation of the CD95 system in RCCs despite upregulation of CD95 receptor and ligand. However, our data clearly demonstrate that the essential components of CD95-mediated signal transduction are available and functionally intact in three out of four RCC cell lines. Thus, we observed a marked increase of apoptosis and a significant ($P < 0.05$) reduction of cell number in all clear cell RCC cell lines upon CD95 receptor activation by *agonistic* anti-CD95 antibodies. Nevertheless, the levels of anticancer drug-induced upregulation of CD95 receptor and ligand were not sufficient for effective activation of CD95-mediated apoptosis in these cell lines.

Interestingly in this context, p53 mutations have recently been found to interfere with anticancer drug-induced and CD95-mediated apoptosis, because wild-type p53 directly transactivates the CD95 receptor (Owen-Schaub et al, 1995; Müller et al, 1998). Consequently, the deficient activation of CD95-mediated cell death in our RCC cell lines could be due to p53 mutations. Although p53 mutations are not very frequent in human RCCs (Reiter et al, 1993), mutant p53 was found in two of our RCC cell lines. In fact, the missing activation of CD95-mediated apoptosis by anticancer drugs might be explained by inadequately low levels of CD95 receptor upregulation in the p53-mutated cell line clearCa-17. In contrast, however, the other p53-mutated RCC cell line (clearCa-6) responded with a marked augmentation of CD95 receptor expression after treatment with anticancer drugs. This observation could indicate the activation of p53-independent signalling pathways for upregulation of CD95 receptors or might be due to functional intactness of p53 despite mutation. The latter hypothesis was supported by the failure to detect nuclear accumulation of p53 protein in clearCa-6 cells by immunocytochemical analysis (data not shown). Thus, nuclear accumulation of p53 protein is an indicator for confirmational changes of p53 protein, interfering with both its function and degradation (Moll and Schramm, 1998). Further evidence for functional intactness of mutant p53 in clearCa-6 could be derived from topotecan-induced upregulation of p21 expression, which is known to be transcriptionally regulated by the p53 gene (El-Deiry et al, 1993; Waldman et al, 1995).

As outlined above, p53 inactivation by mutation could explain the deficient activation of CD95-mediated apoptosis by anticancer drugs in only one of our RCC cell lines. In most RCC cell lines, however, the mechanisms that ultimately determine the response to anticancer drug-induced upregulation of CD95 receptor and ligand are not yet fully understood. This response will critically depend on the balance of all the pro-apoptotic and anti-apoptotic programmes acting along the CD95 signal transduction cascade (for review see Schulze-Osthoff et al, 1998). In this context, it might be of particular relevance that two different types of CD95 signalling pathways have been described only recently, one of which can effectively be blocked by Bcl-2 or Bcl-$x_L$ overexpression (Scaffidi et al, 1998).

Collectively, our results provide convincing evidence that the anticancer drugs topotecan and bleomycin induce upregulation of CD95 receptors and ligands in human RCCs. Despite this upregulation and despite the presence of a functionally intact CD95-signalling cascade, CD95-mediated apoptosis is not effectively activated by these agents. Nevertheless, topotecan and bleomycin exploit alternative pathways also leading to apoptotic cell death. Induction of apoptosis via these alternative pathways, however, is far less effective in human RCCs when compared to the effects of these drugs in other tumour types (Mitsui et al, 1995; Müller et al, 1997). These findings indicate that the deficient activation of CD95-mediated apoptosis by anticancer drugs might additionally contribute to the multidrug resistance phenotype of human RCCs. Therefore, investigations into the mechanisms determining the responsiveness to CD95-mediated apoptosis in human RCCs might eventually facilitate the design of novel therapeutic concepts.

### ACKNOWLEDGEMENTS

We express appreciation to A Florange-Heinrichs, M Bellack, H Auweiler, and M Ringler for excellent technical assistance. Some of the results are part of the PhD thesis of M Dejosez and the medical thesis of B Czarnotta. The work was supported by the Mildred Scheel Stiftung.

### REFERENCES

Baldini N (1997) Multidrug resistance – a multiplex phenomenon. *Nat Med* **3**: 78–380

Caignard A, Guillard M, Cai C, Asselin-Paturel C, Carayol G and Chouaib S (1996) The renal cell carcinoma lysis by a specific cytotoxic T cell clone is independent of the Fas/Fas-L cytotoxic pathway. *Tissue Antigens* **48**: 295–300

Cascino I, Fiucci G, Pappoff G and Ruberti G (1995) Three functional soluble forms of the human apoptosis-inducing Fas molecule are produced by alternative splicing. *J Immunol* **154**: 2700–2713

El-Deiry WS, Tokino T, Velculescu VE, Levy DB, Parsons R, Trent JM, Lin D, Mercer WE, Kinzler KW and Vogelstein B (1993) WAF-1, a potential mediator of p53 tumor suppression. *Cell* **75**: 817–825

Friesen C, Herr I, Krammer PH and Debatin KM (1996) Involvement of the CD95 (APO-1/Fas) receptor/ligand system in drug-induced apoptosis in leukemia cells. *Nat Med* **2**: 574–577

Friesen C, Fulda S and Debatin KM (1997) Deficient activation of the CD95 (APO-1/Fas) system in drug-resistant cells. *Leukemia* **11**: 1833–1841

Fulda S, Sieverts H, Friesen C, Herr I and Debatin KM (1997) The CD95 (APO-1/Fas) system mediates drug-induced apoptosis in neuroblastoma cells. *Cancer Res* **57**: 3823–3829

Fulda S, Los M, Friesen C and Debatin KM (1998) Chemosensitivity of solid tumor cells in vitro is related to activation of the CD95 system. *Int J Cancer* **76**: 105–114

Gerharz CD, Moll R, Störkel S, Ramp U, Thoenes W and Gabbert HE (1993) Ultrastructural appearance and cytoskeletal architecture of the clear cell, chromophilic and chromophobe cell variants of human renal cell carcinoma in vivo and in vitro. *Am J Pathol* **142**: 851–859

Gerharz CD, Ramp U, Olert J, Moll R, Störkel S, Marx N and Gabbert HE (1994) Cytomorphological, cytogenetic, and molecular biological characterization of four new human renal carcinoma cell lines of the clear cell type. *Virchows Arch B* **424**: 403–409

Gerharz CD, Moll R, Störkel S, Ramp U, Hildebrandt B, Molsberger G, Koldovsky P and Gabbert HE (1995) Establishment and characterization of two divergent cell lines derived from a human chromophobe renal cell carcinoma. *Am J Pathol* **146**: 953–962

Gerharz CD, Hilderbrandt B, Moll R, Ramp U, Sarbia M, Störkel S, Koldovsky P and Gabbert HE (1996) Chromophilic renal cell carcinoma: cytomorphological and cytogenetic characterization of four permanent cell lines. *Br J Cancer* **74**: 1605–1614

Harris CC and Hollstein M (1993) Clinical implications of the p53 tumor-suppressor gene. *New Engl J Med* **329**: 1318–1327

Hendricks CB, Rowinsky EK, Grochow LB, Donehower RC and Kaufmann SH (1992) Effect of P-glycoprotein expression on the accumulation and cytotoxicity of topotecan (SK&F 104864), a new camptothecin analogue. *Cancer Res* **52**: 2268–2278

Herben VMM, ten Bokkel Huinink WW and Beijnen JH (1996) Clinical pharmacokinetics of topotecan. *Clin Pharmacokinet* **31**: 85–102

Hetts SW (1998) To die or not to die. An overview of apoptosis and its role in disease. *JAMA* **279**: 300–307

Hickman JA (1992) Apotosis induced by anti-cancer agents. *Cancer Metastasis Rev* **11**: 121–139

Homma Y and Aso Y (1994) Effect of α-Interferon alone and combined with other antineoplastic agents on renal cell carcinoma determined by the tetrazolium microculture assay. *Eur Urol* **25**: 164–170

Horie S, Kano M, Higashihara E, Moriyama N, Tanaka E, Hirose A, Kakizoe T and Kawabe K (1997) Expression of Fas in renal cell carcinoma. *Jpn J Clin Oncol* **27**: 384–388

Keane MM, Ettenberg SA, Lowrey GA, Russel EK and Lipkowitz S (1996) Fas expression and function in normal and malignant breast cell lines. *Cancer Res* **56**: 4791–4798

Kischkel FC, Hellbardt S, Behrmann I, Germer M, Pawlita M, Krammer PH and Peter ME (1995) Cytotoxicity-dependent APO-1 (Fas/CD95)-associated proteins form a death-inducing signalling complex (DISC) with the receptor. *EMBO J* **14**: 5579–5588

Krammer PH (1997) The tumor strikes back. New data on expression of the CD95 (APO-1/Fas) receptor/ligand system may cause paradigm changes in our view on drug treatment and tumor immunology. *Cell Death Differentiation* **4**: 362–364

 *© 2000 Cancer Research Campaign*

Mickisch GH, Bier H, Bergler W, Bak M, Tschada R and Alken P (1990) P-170 glycoprotein, glutathione and associated enzymes in relation to chemoresistance of primary human renal cell carcinomas. *Urol Int* **45**: 170–176

Mitsui I, Kumazawa E, Hirota Y, Aonuma M, Sugimori M, Ohsuki S, Uoto K, Ejima A, Terasawa H and Sato K (1995) A new water-soluble camptothecin derivate, DX-8951f, exhibits potent antitumor activity against human tumors in vitro and in vivo. *Jpn J Cancer Res* **86**: 776–782

Miyake H, Hara I, Gohji K, Arakawa S and Kamidono S (1998) p53 modulation of Fas/APO-1 mediated apoptosis in a human renal cell carcinoma cell line. *Int J Oncol* **12**: 469–473

Moll UM and Schramm LM (1998) p53 – an acrobat in tumorigenesis. *Crit Rev Oral Biol Med* **9**: 23–37

Mosmann T (1983) Rapid colorimetric assay for cellular growth and survival: application to proliferation and cytotoxicity assays. *J Immunol Methods* **65**: 55–64

Mulders P, Figlin R, deKernion JB, Wiltrout R, Linehan M, Parkinson D, deWolf W and Belldegrun A (1997) Renal cell carcinoma: recent progress and future directions. *Cancer Res* **57**: 5189–5195

Müller M, Strand S, Hug H, Heinemann EM, Walczak H, Hofmann WJ, Stremmel W, Krammer PH and Galle PR (1997) Drug-induced apoptosis in hepatoma cells is mediated by the CD95 (APO-1/Fas) receptor/ligand system and involves activation of wild-type p53. *J Clin Invest* **99**: 403–413

Müller M, Wilder S, Bannasch D, Israeli D, Lehlbach K, Li-Weber M, Friedman SL, Galle PR, Stremmel W, Oren M and Krammer PH (1998) p53 activates the CD95 (APO-1/Fas) gene in response to DNA damage by anticancer drugs. *J Exp Med* **11**: 2033–2045

Nagata S (1994) Apoptosis regulated by a death factor and its receptor: Fas ligand and Fas. *Philos Trans R Soc Lond B Biol Sci* **345**: 281–287

Nakajima H, Yamada N and Takiguchi M (1998) Fas-independent apoptosis of T cells via killer cell inhibitory receptors. *Int Immunol* **10**: 85–90

Natoli G, Ianni A, Constanzo A, De Petrillo G, Ilari I, Chirillo P, Balsano C and Levrero M (1995) Resistance to Fas-mediated apoptosis in human hepatoma cells. *Oncogene* **11**: 1157–1164

Nonomura N, Miki T, Yokoyama M, Imazu T, Takada T, Takeuchi S, Kanno N, Nihimura K, Kojima Y and Okuyama A (1996) Fas/APO-1-mediated apoptosis of human renal cell carcinoma. *Biochem Biophys Res Commun* **229**: 945–951

O'Connell J, O'Sullivan GC, Collins JK and Shanahan F (1996) The Fas counterattack: Fas-mediated T cell killing by colon cancer cells expressing Fas ligand. *J Exp Med* **184**: 1075–1082

Owen-Schaub LB, Zhang W, Cusack JC, Angelo LS, Santee SM, Fujiwara T, Roth JA, Deisseroth AB, Zhang WW, Kruzel E and Radinsky R (1995) Wild-type human p53 and a temperature-sensitive mutant induce FAS/APO-1 expression. *Mol Cell Biol* **15**: 3032–3040.

Peter ME, Dhen I, Ehret A, Helbardt S, Walczak H, Moldenhauer G and Krammer PH (1995) APO-1 (CD95)-dependent and independent antigen-receptor-induced apoptosis in human T and B cell lines. *Int Immunol* **7**: 1873–1877

Ramp U, Jaquet K, Reinecke P, Nitsch T, Gabbert HE and Gerharz CD (1997) Acquisition of TGF-$\beta_1$ resistance: an important progression factor in human renal cell carcinoma. *Lab Invest* **76**: 739–749

Reiter RE, Anglard P, Liu S, Gnarra JR and Linnehan WM (1993) Chromosome 17p deletions and p53 mutations in renal cell carcinoma. *Cancer Res* **53**: 3092–3097

Scaffidi C, Fulda S, Srinivasan A, Friesen C, Li F, Tomaselli KJ, Debatin KM, Krammer PH and Peter ME (1998) Two CD95 (APO-1/Fas) signalling pathways. *EMBO J* **17**: 1675–1687

Scheithauer W, Clark GM, Salmon SE, Dorda W, Shoemaker RH and von Hoff DD (1986) Model for estimation of clinically achievable plasma concentrations for investigational anticancer drugs in man. *Cancer Treatment Reports* **70**: 1379–1382

Schulze-Osthoff K, Ferrari D, Los M, Wesselborg S and Peter ME (1998) Apoptosis signalling by death receptors. *Eur J Biochem* **254**: 439–459

Sorensen M, Sehested M and Jensen PB (1995) Characterisation of a human small-cell lung cancer cell line resistant to the DNA topoisomerase I-directed drug topotecan. *Br J Cancer* **72**: 399–404

Strand S, Hofmann WJ, Hug H, Müller M, Otto G, Strand D, Mariani SM, Stremmel W, Krammer PH and Galle PR (1996) Lymphocyte apoptosis induced by CD95 (APO-1/Fas) ligand-expressing tumor cells – a mechanism of immune evasion? *Nat Med* **2**: 1361–1366

Tomita Y, Kawasaki T, Bilim V, Takeda M and Takahashi K (1996) Tetrapeptide DEVD-aldehyde or YVAD-chloromethylketone inhibits Fas/APO-1 (CD95)-mediated apoptosis in renal cancer cells. *Int J Cancer* **68**: 132–135

Waldman T, Kinzler KW and Vogelstein B (1995) p21 is necessary for the p53-mediated G1 arrest in human cancer cells. *Cancer Res* **55**: 5187–5190

Webb JL (1963) Effects of more than one inhibitor. In: *Enzyme and Metabolic Inhibitors*. Vol 1. pp. 487–512. Academic Press: New York.

*© 2000 Cancer Research Campaign*
